# Supplementary material for: Sustainability analysis framework for on-demand public transit systems
Source: Sci Rep. 2023 Aug 18;13:13488. doi: 10.1038/s41598-023-40639-y (PMC10439202; doi:10.1038/s41598-023-40639-y)
Supplement: Supplementary file 1 — Supplementary Information. [file 41598_2023_40639_MOESM1_ESM.pdf]

Supplementary Information for: Sustainability Analysis Framework for On-Demand Public Transit Systems

Nael Alsaleh[1] **and Bilal Farooq**[1,*]

[1]Laboratory of Innovations in Transportation (LiTrans), Department of Civil Engineering, Toronto Metropolitan University, Toronto, ON M5B 2K3, Canada
[*]bilal.farooq@torontomu.ca

Supplementary Tables

**Results**		**Waiting Time (min)**	**In-Vehicle Time (min)**	**Trip Length (km)**
Actual	Average	8.12	10.22	9.28
	St.Dev	5.17	6.65	4.11
Simulated	Average	8.72	11.39	9.76
	St.Dev	5.91	6.73	4.33
Difference	Average	-0.60	1.17	0.48
	St.Dev	7.52	8.68	5.19
	t-value	1.04*	1.79*	1.23*

*Not significant at 95% confidence level.

Supplementary Table 1. Statistical difference between actual and simulated performance. The comparison showed no statistical difference between the simulation results and the actual data, in terms of in-vehicle travel times, trip length, and waiting time values, at 95% confidence level.

Supplementary Figures

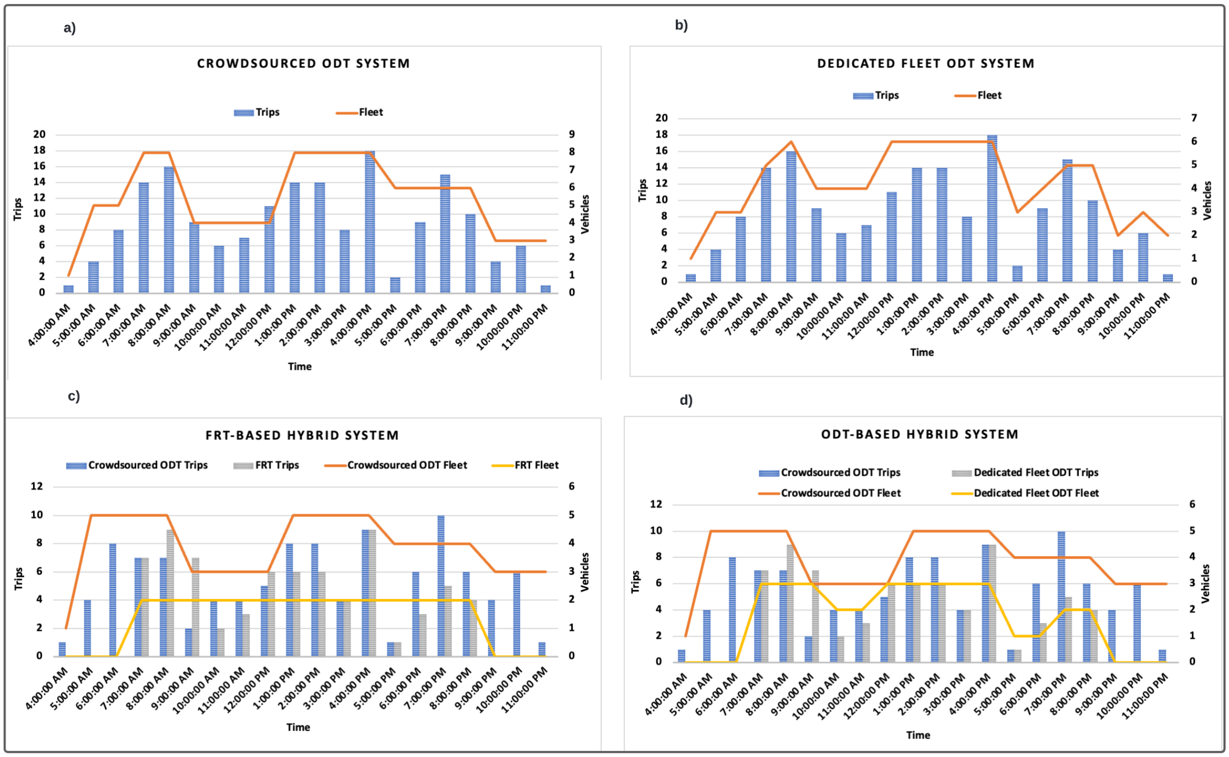

Supplementary Figure 1. The temporal distribution of demand and supply for a) the crowdsourced ODT system, b) dedicated fleet ODT system, c) the FRT-based hybrid system, and d) ODT-based hybrid system.

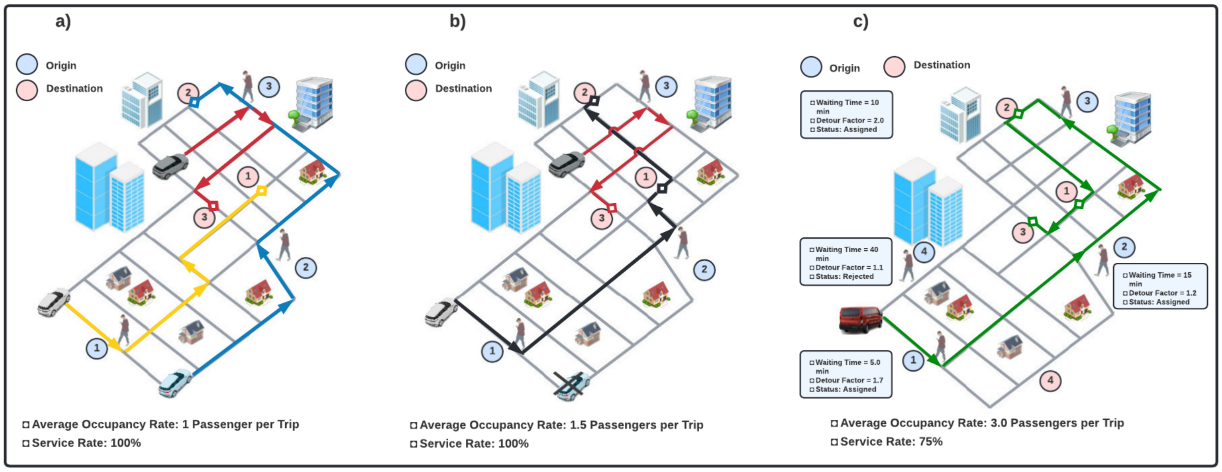

Supplementary Figure 2. Differences between the dispatching algorithms used in the crowdsourced ODT and the dedicated fleet ODT scenarios. a) crowdsourced ODT service with exclusive-rides. b) crowdsourced ODT service with shared-rides. c) Dedicated fleet ODT service.

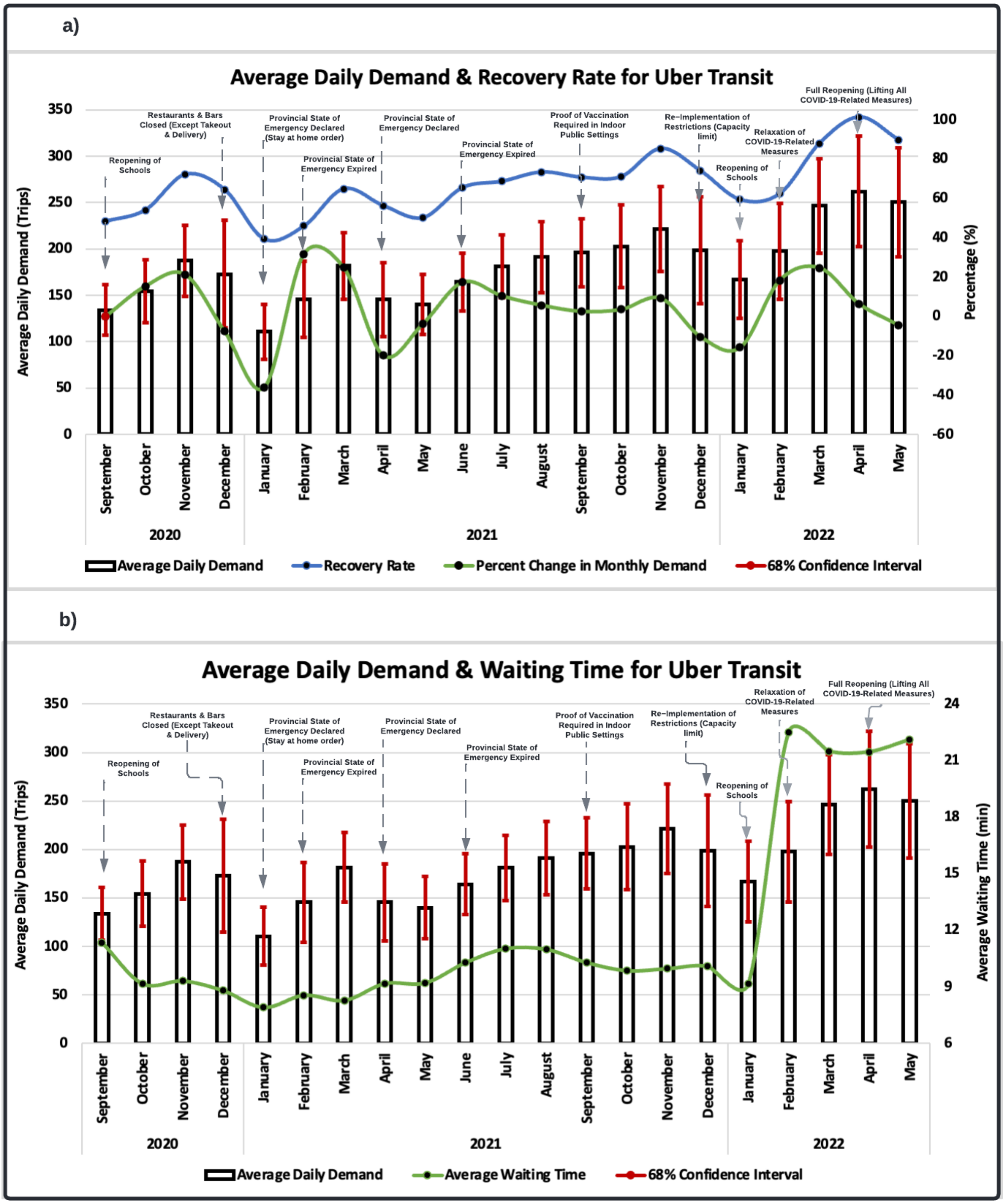

Supplementary Figure 3. The impact of the COVID-19 pandemic on the average daily demand and waiting time for the existing crowdsourced ODT in Innisfil. Demand recovery rates are calculated based on the 2019 demand levels. It is worth noting that the analysis was done on the data set from September 2020 in line with the effective start date of the data-sharing agreement between Innisfil and Uber Technologies Inc.

Supplementary Notes

Supplementary Fig. 3 presents the average daily demand and waiting time for the service from September 2020 until May 2022. It is observed that the service experienced three cycles of a sharp decrease in demand followed by a gradual recovery. The highest reductions in demand were recorded in January and April 2021, when the province declared a stay-at-home order, at 58% and 41% of the 2019 levels, respectively. However, the service resumed its 2019 demand in April 2022 after all COVID-19 restrictions were lifted. On the other hand, the average daily waiting time values shown in Supplementary Fig. 3b illustrate the relationship between demand and supply. From September 2020 to January 2022, supply followed demand, rising and falling, resulting in consistent wait times between 8 and 11 minutes. In February 2022, the demand started to increase as a result of the relaxation of COVID-19 related restrictions. The increase in demand, however, was not accompanied by an adequate rise in supply, which led to a dramatic increase in the average waiting time. In March 2022, the Town of Innisfil launched an incentive program, which resulted in a slight decrease in waiting times in the following months.
